# Knowledge, attitudes, perceptions, and COVID-19 hesitancy in a large public university in Mexico city during the early vaccination rollout

**DOI:** 10.1186/s12889-022-14225-2

**Published:** 2022-10-04

**Authors:** Norma Mongua-Rodríguez, Mauricio Rodríguez-Álvarez, Daniela De-la-Rosa-Zamboni, María Eugenia Jiménez-Corona, Martha Lucía Castañeda-Cediel, Guadalupe Miranda-Novales, Gustavo Cruz-Pacheco, Elizabeth Ferreira-Guerrero, Leticia Ferreyra-Reyes, Guadalupe Delgado-Sánchez, Maribel Martínez-Hernández, Arturo Cruz-Salgado, Rogelio Pérez-Padilla, Samuel Ponce-de-León, Lourdes García-García

**Affiliations:** 1 Instituto Nacional de Salud Pública, Cuernavaca, Morelos México; 2 Programa Universitario de Investigación en Salud, Universidad Nacional Autónoma de México Ciudad Universitaria, Ciudad de México, México; 3 Hospital Infantil de México “Federico Gómez”, Ciudad de México, México; 4 Instituto Nacional de Cardiología, Ciudad de México, México; 5 Posgrado en Geografía, Universidad Nacional Autónoma de México, Ciudad Universitaria, Ciudad de México, México; 6 Universidad Nacional Autónoma de México, Ciudad de México, México; 7 Instituto de Investigaciones en Matemáticas Aplicadas y en Sistemas, Universidad Nacional Autónoma de México, Ciudad de México, México; 8 Instituto Nacional de Enfermedades Respiratorias, Ciudad de México, México; 9 Instituto Nacional de Salud Pública, Av. Universidad # 655, Col. Sta. María Ahuacatitlán, C.P. 62100 Cuernavaca, Morelos México

**Keywords:** Vaccine, COVID-19, SARS-CoV-2, Survey, Knowledge, Attitudes, Practices, University, Mexico

## Abstract

**Background:**

Vaccination against COVID-19 is a primary tool for controlling the pandemic. However, the spread of vaccine hesitancy constitutes a significant threat to reverse progress in preventing the disease. Studies conducted in Mexico have revealed that vaccination intention in Mexico among the general population ranges from 62 to 82%.

**Objective:**

To know the prevalence of COVID-19 vaccine hesitancy and associated factors among academics, students, and administrative personnel of a public university in Mexico City.

**Methods:**

We administered an online survey investigating sociodemographic aspects, knowledge, attitudes, practices, and acceptance/hesitancy regarding the COVID-19 vaccine. Using generalized linear Poisson models, we analyzed factors associated with vaccine hesitancy, defined as not intending to be vaccinated within the following six months or refusing vaccination.

**Results:**

During May and June 2021, we studied 840 people, prevalence of vaccine hesitancy was 6%. Hesitancy was significantly associated with fear of adverse effects, distrust of physician’s recommendations, lack of knowledge regarding handwashing, age younger than 40 years, refusal to use face masks, and not having received influenza vaccination during the two previous seasons.

**Conclusions:**

Vaccine hesitancy in this population is low. Furthermore, our results allowed us the identification of characteristics that can improve vaccine promotion.

## Background

From its appearance in December 2019 to November 2021, SARS-CoV-2 has caused more than 245 million cases of coronavirus disease 2019 (COVID-19) and 5 million deaths worldwide [1]. Up to the end of 2021, the impact of COVID-19 in Mexico had been devastating, with mortality rates (2,256.6 deaths per million inhabitants) and excess mortality (41.45% excess deaths from all causes compared to projection based on previous years) that ranked 24th and 5th among 210 countries [1].

The World Health Organization considers vaccination one of the most cost-effective ways of preventing disease. It currently prevents 2–3 million deaths a year, and a further 1.5 million could be avoided if global coverage of vaccinations improved [2]. Up to February 2021, more than 50 COVID-19 vaccine candidates had been developed [3]. The efficacy of the different vaccines has ranged from 50% to 95% against symptomatic COVID-19. This has resulted in more than one hundred countries approving or authorizing the emergency use of these vaccines between December 2020 and February 2021 [1].

Based on WHO guidelines, Mexico’s Covid-19 Vaccine Technical Advisory Group issued recommendations for Covid vaccination based on groups that would receive the most significant benefit of immunization, initially prioritizing health workers and people aged 60 years or older with or without comorbidities, people aged 50 to 59 years with comorbidities subsequently descending every decade of life, to continue the vaccination in the remaining population [4]. Up to the submission of this study, none of the vaccines available in Mexico could be used in people under 12 years of age, for which a specific stage was not yet contemplated for this population. SARS-Cov-2 vaccination was initiated in December 2020. Up to March 2022, the regulatory agency in Mexico (Federal Commission of Protection of Sanitary Risks, COFEPRIS) has agreements with the following pharmaceutical companies: Pfizer-BioNTech, Cansino, COVAX, AstraZeneca, Sputnik V, Sinovac, Janssen, and Moderna. COVID-19 vaccination in Mexico has been free and voluntary [5]. By November 2021, 58.9% of the Mexican population had been vaccinated with at least one dose of the COVID-19 vaccine [1].

The challenge to achieve high vaccination coverage has required political will, equity, solidarity, and rigorous planning for production, purchase, reception, and storage. However, the success of any vaccine depends on the proportion of the population that gets vaccinated. The SAGE Working Group on Vaccination Hesitancy was an important forum established in 2012 to map the determinants of vaccination hesitancy and recommend strategies to address what was finally recognized as a growing problem. This group concluded that vaccine hesitancy refers to delayed acceptance or refusal of vaccination despite the availability of vaccination services. Vaccine hesitancy is complex and context-specific, varying by time, place, and vaccines. It is influenced by factors such as complacency, convenience, and trust [6].

Several reviews have shown a wide variety of COVID-19 vaccine hesitancy worldwide. In a review of 82 studies, Shakeel et al. revealed variations in vaccine acceptance among adults ranging from 27.0% in the Republic of Congo to 97% in Ecuador. Low vaccine acceptance was associated with low education and awareness levels and inefficient government efforts and initiatives. Furthermore, poor influenza-vaccination history, conspiracy theories relating to infertility, and misinformation about the COVID-19 vaccine on social media also resulted in vaccine hesitancy [7]. Another review analyzed surveys in 114 countries/territories. COVID-19 vaccine acceptance rates ≥ 60% were seen in 72/114 countries/territories, compared to 42 countries/territories with rates between 13% and 59%. COVID-19 vaccine hesitancy appeared more pronounced in the Middle East/North Africa, Europe and Central Asia, and Western/Central Africa [8]. Nehal et al. included 63 studies in 30 countries, finding that global acceptance of the vaccine was 66.0% [9]. Focusing on low- and lower-middle-income countries, Patwary et al. reviewed 36 studies, including 83,867 respondents. The pooled-effect size of the COVID-19 vaccine hesitancy rate was 38.2%. Being male and perceiving the risk of COVID-19 infection were predictors of willingness to accept the vaccine [10]. Bono et al. studied 10,491 participants in 83 low- and middle-income countries, with results showing that acceptance was 88.8% overall, higher in Brazil, the only American country included in the study, than in other Asian and African countries [11]. Urrunaga et al. studied 472,521 participants in Latin America, with 80% intention to vaccinate, with Mexico ranking higher with 88.4% [12]. Mexican studies have examined the general population, among whom vaccination intention ranges from 62 to 82% [12–14]. We hypothesized that COVID-19 hesitancy among academics, students, and administrative personnel of a public university in Mexico City would be lower than among the general population. We also aimed to analyze hesitancy-associated factors.

## Methodology

We administered an online survey to students, faculty, and administrative personnel of the National Autonomous University in Mexico. Participation was anonymous and voluntary. Participants completed an online survey between May 20 and June 27, 2021.

The survey was available on a virtual platform on the university web page and shared in e-mails and social networks [15].

We used convenience sampling, including only people over 18 years of age, with the capacity to answer a self-administered questionnaire and who voluntarily agreed to participate.

The survey was designed by a multidisciplinary research team (infectious disease specialists, epidemiologists, public health specialists, communicators, designers, and computer scientists). The instrument was piloted among university workers, and a panel of experts reviewed the items and questions.

We followed the recommendations of the Sage Working Group on Vaccine Hesitancy, which grouped the factors influencing vaccine hesitancy into three categories: contextual, individual, and group, and vaccine/vaccination-specific influences [6]. We aimed to identify the following aspects that promote vaccine hesitancy or acceptance: demographic factors, personal responsibility and risk perceptions, preventive measures based on the perceived risk, trust in health authorities and vaccines, safety and efficacy of a new vaccine, and lack of information or vaccine misinformation [16]. The survey consisted of 23 questions that included socio-demographic aspects of the participant (age, sex, educational level, trade or job, perception of remuneration for the work performed); status of contacts concerning COVID-19; comorbidities (DM, hypertension, or obesity), knowledge about COVID-19; attitudes or perceptions towards COVID and self-care practices including the intention to be vaccinated and the reasons involved in the decision to get vaccinated. These questions were presented with dichotomous, multiple-choice, or Likert-scale answer options. Questions varied in format, but most used a 5-point Likert response scale ranging from strongly agree to disagree strongly (Cronbach α range, 0.75).

The following questions explored vaccine hesitancy/acceptance: “Would you get the COVID-19 vaccine?(answer options: Yes, No, I don’t know, I already got vaccinated) and “When would you get vaccinated against COVID-19? ″ (Answer options: immediately; between 1 and 6 months; between 6 months and one year; between 1 and 2 years; after two years; never; other). Participants were hesitant when they answered that they did not want to be vaccinated, were doubtful, or would delay vaccination for more than six months. Vaccine acceptance was considered if the participant had already been vaccinated, was willing to get vaccinated, and intended to immediately receive the vaccine as soon as it was available for their age group.

We included participants who answered questions regarding their intention to be vaccinated and provided a valid zip code. We compared the characteristics of participants with those of non-participants.

Responses regarding knowledge about COVID-19; attitudes or perceptions towards COVID, and self-care practices were categorized into two groups: Those who agreed (“total agreement” or “partially agreement”) and those who disagreed (“neither agreeing nor disagreeing,” “partially disagreeing,” “totally disagreeing,” and “does not know” or “does not answer”). Participants’ characteristics are presented as mean (standard deviation (SD) for continuous measures with normal distribution, while categorical variables are presented as absolute (No) and relative (%) frequencies. We used the chi-square or Mann-Whitney test to compare participant characteristics according to hesitancy/acceptance. We constructed generalized linear Poisson models to investigate vaccine hesitancy/acceptance variables. Variables were entered into the models according to their statistical significance in the bivariate analysis (p ≤ 0.2) and their biological relevance and were retained based on the x2 test of the log-likelihood ratios. A two-sided P value of less than 0.05 indicates statistical significance in all analyses[17]. Statistical analysis was done using the STATA statistical package version 15.

## Results

**Fig. 1:**
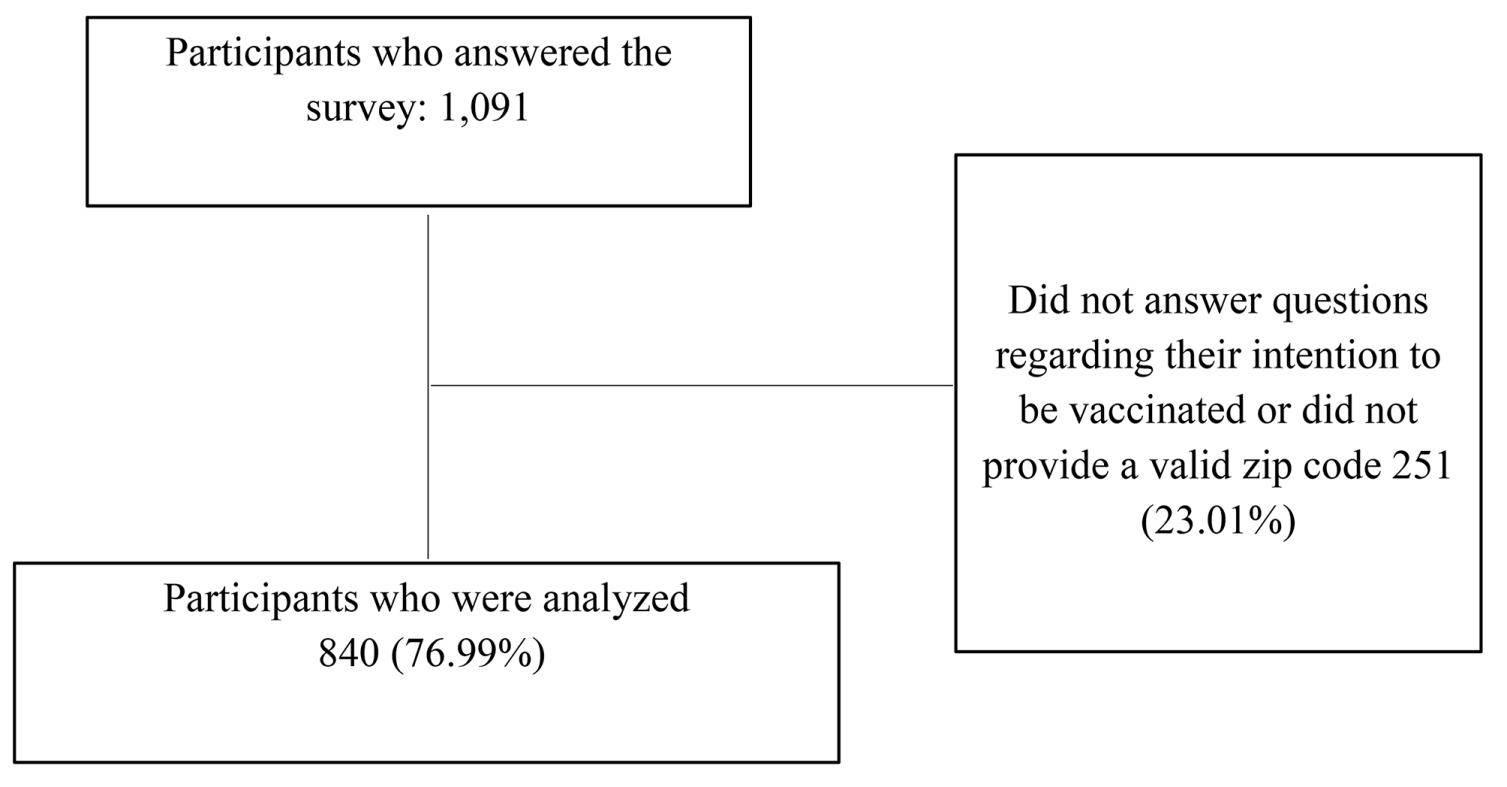
Flowchart of participants, survey on intention to vaccinate against COVID-19-UNAM 2021

One thousand ninety-one participants submitted their questionnaire, of whom 182 (16.68%) did not provide information about their zip code, and 69 (6.32%) did not answer questions about their intent to be vaccinated. Therefore, we analyzed 840 (76.99%) participants. Most lived in Mexico City (70.41%) and the State of Mexico (11.88%). (Fig. 1).

A comparison of the study population with those that did not provide complete information revealed that there were no statistical differences regarding sex, age group, formal education, type of occupation, comorbidities (DM, hypertension, or obesity), history of influenza vaccination in two previous seasons and history of tetanus vaccination. Individuals who referred to having a paid job or who had been in contact with a person ill or dead from COVID-19 were more likely to send complete information (Table 1).

**Table 1:** Characteristics of the studied population compared to those who did not accept participation or with incomplete information

Characteristics	Studied		Not Studied		P-value*
	**No (840)**	**% (76.99)**	**No (251)**	**% (23.01)**	
Male	268/839	31.94	46/168	27.38	0.244
40 years or more	595/840	70.83	108/153	70.59	0.951
Undergraduate	52/840	6.19	14/126	11.11	0.108
Graduate	352/840	41.90	47/126	37.30	
Postgraduate	436/840	51.90	65/126	51.59	
Paid work	693/840	82.50	95/251	37.85	< 0.001
Employee	528/836	63.16	52/98	53.06	0.205
Student	12/836	1.44	3/98	3.06	
Independent without employees	101/836	12.08	18/98	18.37	
Employer	48/836	5.74	7/98	7.14	
Unemployed	147/836	17.58	18/98	18.37	
COVID-19 in contacts	790/840	94.05	91/251	36.25	< 0.001
Death (s) due to COVID-19 in contacts	677/840	80.60	70/251	27.89	< 0.001
DM, hypertension, or obesity	352/840	41.90	41/88	46.59	0.397
Was vaccinated against influenza (2019–2020)	474/840	56.43	24/ 48	50.00	0.383
Was vaccinated against influenza (2020–2021)	477/840	56.79	25/46	54.35	0.745
Tetanus vaccine in the last five years	230/840	27.38	10 /49	20.41	0.060
Tetanus vaccine more than five years ago	271/840	32.26	16/49	32.65	
Has never received tetanus vaccine	35/840	4.17	6 /49	12.24	
Does not remember receiving the tetanus vaccine	304/840	36.19	17/49	34.69	

* *x*^*2*^ test, No=number

Almost 6% of the participants were hesitant about receiving the vaccine. Crude analyses showed that vaccine hesitancy was associated with younger age, fewer years of formal education, unpaid work, having suffered from Covid-19, not having been diagnosed with hypertension, and not having received influenza vaccine during the two previous winter seasons. Table 2.

**Table 2:** Characteristics of the surveyed population on intention to be vaccinated against COVID-19

Characteristics	Hesitant		Intends to be vaccinated or vaccinated		P-value^*^
	**No (50)**	**% (5.95)**	**No (790)**	**% (94.05)**	
Male	16	32.00	252	31.90	0.969
40 years or older	29	58.00	566	71.65	0.040
Undergraduate	6	12.00	46	5.82	0.034
Graduate	26	52.00	326	41.27	
Postgraduate	18	36.00	418	52.91	
Paid work	35	70.00	658	83.29	0.016
Employee	26	52.00	502	63.54	0.120
Student	0	0.00	12	1.52	
Independent without employees	8	16.00	93	11.77	
Employer	1	2.00	47	5.95	
Unemployed	15	30.00	132	16.71	
Has suffered COVID-19	14	28.00	114	14.43	0.010
COVID-19 in a relative living in the same home	11	22.00	139	17.59	0.430
COVID-19 in a relative living in a different home	28	56.00	501	63.42	0.292
COVID-19 in a neighbor	18	36.00	287	36.33	0.963
COVID-19 in friend or acquaintance	31	62.00	527	66.71	0.494
Death(s) due to COVID-19 in a relative living in the same home	2	4.00	15	1.90	0.306
Death(s) due to COVID-19 in a relative living in a different home	11	22.00	277	35.06	0.059
Death(s) due to COVID-19 in a friend or acquaintance	26	52.00	485	61.39	0.187
Death(s) due to COVID-19 in a neighbor	15	30.00	182	23.04	0.260
Death(s) due to COVID-19 in an acquaintance	12	24.00	138	17.47	0.242
Diabetes	4	8.00	53	6.71	0.265
Hypertension	4	8.00	117	14.81	< 0.001
Overweight or obesity	14	28.00	277	35.06	0.596
Was vaccinated against influenza (2019–2020)	18	36.00	456	57.72	0.003
Was vaccinated against influenza (2020–2021)	15	30.00	462	58.48	< 0.001
Was vaccinated against tetanus in the last five years	11	22.00	219	27.72	0.109
Was vaccinated against tetanus more than five years ago	13	26.00	258	32.66	
Has never received tetanus vaccine	5	10.00	30	3.80	
Does not remember receiving the tetanus vaccine	21	42.00	283	35.82	

* *x*^*2*^ test; No = number.

Hesitant participants were more likely to lack knowledge regarding the transmissible nature of the disease or the severity of COVID-19 among people with comorbidities, were less afraid of falling ill, were uncomfortable or ashamed when wearing a facemask, distrusted physician or family´s recommendations did not believe that the vaccine is useful or considered immunization unnecessary, feared adverse effects, referred having had bad experiences with other vaccines, would never vaccinate their children and refused to use facemasks, Table 3.

**Table 3:** Knowledge, attitudes, and perceptions of the study population about the COVID-19 vaccine

Characteristic	Hesitant (No = 50)	Intending to be vaccinated or vaccinated (No = 790)	P-value*
Knows that a virus causes COVID-19. (No. (%)	46 (92.00)	754 (95.40)	0.268
Knows that SARS-CoV-2 is transmissible. (No. (%)	49 (98.00)	790 (100)	< 0.001
Knows that washing hands prevents COVID-19. (No. (%)	48 (96.00)	782 (98.99)	0.059
Knows that people with comorbidities have a worse prognosis. (No. (%)	47 (94.00)	780 (98.73)	0.009
Knows that face masks prevent COVID-19. (No. (%)	47 (94.00)	765 (96.84)	0.279
Is concerned about falling ill. (No. (%)	41(82.00)	747 (94.56)	< 0.001
Experiences discomfort when wearing a mask. (No. (%)	27 (54.00)	312 (39.49)	0.043
Is ashamed when wearing a mask. (No. (%)	3 (6.00)	5 (0.63)	< 0.001
Trusts physician’s recommendations. (No. (%)	31 (64.58)	739 (94.74)	< 0.001
Trusts family’s recommendations. (No. (%)	14 (29.17)	386 (49.49)	0.006
Trusts friends’ recommendations. (No. (%)	14 (29.17)	347 (44.49)	0.038
Fearful of adverse effects from the vaccine. (No. (%)	6 (12.50)	17 (2.18)	< 0.001
Disbelief in the usefulness of the COVID-19 vaccine. (No. (%)	3 (6.25)	9 (1.15)	0.004
Considers that the COVID-19 vaccine is unnecessary. (No. (%)	8 (1.03)	6 (12.50)	< 0.001
Fear of adverse effects. (No. (%)	34 (70.83)	305 (39.10)	< 0.001
Has had bad experiences with other vaccines. (No. (%)	18 (37.50)	190 (24.36)	0.042
Will never vaccinate their children. (No. (%)	8 (16.67)	12 (1.54)	< 0.001
Refuses to use face masks. (No. (%)	1 (2.00)	2 (0.25)	0.045

* *x*^*2*^ test, No = number.

Adjusted analysis showed that vaccine hesitancy was significantly associated with fear of adverse effects, distrust of physician’s recommendations, lack of knowledge regarding handwashing, age younger than 40 years, refusal to use face masks, and not having received influenza vaccination during the two previous seasons, Table 4.

**Table 4:** Factors associated with COVID-19 vaccine hesitancy

Characteristic/Attitude/Condition	PR	CI 95%	P-value*
Fear of adverse effects	4.42	2.58–7.56	< 0.001
Distrusts physician’s recommendations	3.99	2.27–6.99	< 0.001
Does not know that washing hands prevents COVID-19	3.64	1.84–7.21	< 0.001
***Socio-demographic***			
Under 40 years of age	2.15	1.30–3.55	0.003
40 years of age or older	Ref	-	-
Refuses to use face masks	2.11	1.29–3.43	0.001
No 2019–2020 influenza vaccination	2.08	1.14–3.79	0.017
No person has died due to COVID-19 among family members, neighbors, or acquaintances	1.39	0.85–2.28	0.196

*Generalized Poisson linear model; PR = Prevalence Ratio.

## Discussion

The results of our study highlight the high acceptance rates (96%) of vaccination among academics, students, and administrative personnel of a public university in Mexico City.

Our findings contrast with two nationally representative surveys conducted at the end of 2020 among Mexican adults that showed COVID-19 vaccine acceptance of 62.3% and 82%, respectively [13, 18]. The higher acceptance rates in our study conducted almost six months later may be due to increasing trends of vaccine acceptance over time after intense vaccine promotion campaigns and high hospitalization and mortality rates due to COVID-19. Different studies have described changes in acceptance or rejection trends over time. Sallam et al. reported that acceptance increased from 56.9% in April 2020 to 75.4% in June in the United States [19]. On the other hand, the survey of several European countries by Neumann-Böhme et al. reported a 20% decrease in acceptance during their study period [20]. As described previously for the Mexican population [13], we found that unpaid work and fewer years of formal education were associated with COVID-19 vaccine hesitancy by bivariate analysis.

Our definition of hesitancy allowed us to encompass individuals who refuse vaccination and those who have doubts about being vaccinated in the short term. This definition enables better feedback for public policies since it considers factors related to trust, perceived effectiveness, and convenience [6]. Diverse studies suggest that the decision to be vaccinated varies according to the disease, the type of vaccine, the target population (age, level of schooling, race, ethnicity), the particular socio-economic context, the country´s income, the existence of legal regulations regarding vaccination, vaccine access and professional information [21–23]; additionally, different definitions of vaccine acceptance, hesitancy, and refusal influence the results.

An advantage of our study was that we explored reasons for hesitancy. We found that the main reason for vaccine hesitancy was fear of adverse effects. This factor has been widely described in the literature [11, 12, 22–24].

Our results underline the importance that the population links to the recommendation of the medical personnel. Participants who distrusted physicians’ recommendations were nearly four times more likely to refuse the vaccine. Nehal et al. described that vaccine guidance was relevant, both from the members of participants’ social networks and those of their physicians [9]. According to several authors, trust refers to the attitude towards the vaccine advice from health personnel or community leaders [12]. Different studies have documented the usefulness of the person-centered health care model in changing behaviors and attitudes. The benefit of the model is based on health personnel’s willingness to listen and privilege the preferences, needs, and individual values of the person who consults them [6].

The population that most likely refused the vaccine had not been vaccinated against influenza in previous seasons, a finding described by other researchers [23, 25]. Vaccine hesitancy was also linked to not knowing that handwashing prevents infection or refusal to use facemasks. We consider that these variables may be indicators of individual self-efficacy and the perceived effectiveness of health services. This path provides individuals with the necessary information and security to be active participants in decisions that concern their health. From a more general point of view, the vaccination process must consider political, economic, cultural, social, and religious contexts [26].

Other studies have found an association between vaccine rejection with age, sex, and socioeconomic level [9, 11–14, 22, 23, 27]. The direction of the association has been variable. These findings underline the complexity of vaccine determinants. As reported by other authors, our work detected that only the youngest age was associated with hesitancy among sociodemographic factors [11]. When adjusting the model for the significant or relevant variables, we did not find other demographic or clinical characteristics related to vaccine rejection.

Our study has several limitations. We followed the convenience and “snowball” methodology, so it does not necessarily represent the entire population sharing the same characteristics. Our study invited only academics, students, and administrative personnel of a public university with internet access; therefore, our results are not generalizable to the population who lacked this connection. By comparing the features of individuals who completed the survey with those who did not complete it, we found that those who had suffered from COVID-19 or had family members or acquaintances who had presented or died from this cause were more likely to complete it. Secondly, it was a cross-sectional study, so we do not know if there were changes in acceptance or rejection over time.

## Conclusion

In contrast to previous studies conducted globally and in Latin America, our results revealed a low prevalence of vaccine hesitancy (6%). Hesitancy was significantly associated with fear of adverse effects, distrust of physician’s recommendations, lack of knowledge regarding handwashing, age younger than 40 years, refusal to use face masks, and not having received influenza vaccination during the two previous seasons. Vaccine promotion should improve communication with younger people, provide accurate information on adverse effects, and train health personnel for effective messaging.

## Data Availability

Data and materials are available in the manuscript and tables
